# Dietary Hydroxy-Selenomethionine Improves Antioxidant Status and Reduces Somatic Cell Count in Dairy Cows: Multi-Omics Insights into Rumen Microbiota and Metabolic Profiles

**DOI:** 10.3390/antiox15070813

**Published:** 2026-06-28

**Authors:** Jiaxuan Song, Guanghuan Kong, Xinling Wang, Yunfei Zhai, Jiajie Wang, Jie Xu, Chongjun Li, Wudong Liu, Yaodi Han, Zhaoyu Han

**Affiliations:** 1 College of Animal Science and Technology, Nanjing Agricultural University, Nanjing 210095, China; 2024805106@stu.njau.edu.cn (J.S.); 2022205004@stu.njau.edu.cn (Y.Z.); 2023805109@stu.njau.edu.cn (J.W.); 2025205011@stu.njau.edu.cn (J.X.); 2024205012@stu.njau.edu.cn (C.L.); 2024105008@stu.njau.edu.cn (W.L.); 2024805105@stu.njau.edu.cn (Y.H.); 2 Pudong New Area Rural and Agricultural Affairs Commission, Shanghai 201299, China; lovelccyes@163.com; 3 College of Animal Science and Food Engineering, Jinling Institute of Technology, Nanjing 210038, China; xinling@jit.edu.cn

**Keywords:** hydroxy-selenomethionine, dairy cow, antioxidant, 16S rRNA sequencing, metabolomics

## Abstract

High-yielding dairy cows are highly susceptible to lactational oxidative stress, which compromises mammary barrier integrity and elevates mastitis risk. This study investigated the potential biological mechanisms by which dietary hydroxy-selenomethionine (HMSeBA) alleviates oxidative stress and improves health in dairy cows. Forty Holstein cows were assigned to a basal control group (0.32 mg Se/kg DM) or an HMSeBA-supplemented group (0.64 mg Se/kg DM) for 105 days. HMSeBA significantly enhanced selenium bioavailability in both milk and blood, comprehensively strengthening antioxidant defenses (increased glutathione peroxidase activity, decreased malondialdehyde) and elevated serum immunoglobulins (IgA, IgM, IgG), accompanied by a reduction in milk somatic cell count, without significantly affecting milk yield, feed intake, or milk production efficiency. Multi-omics analysis revealed that HMSeBA supplementation altered the rumen microenvironment by enriching fiber-degrading genera (*Prevotellaceae*_Ga6A1_group, *Xylanibacter*, *Segatella*) and shifting metabolites, including feed flavonoids, peptides, 1-deoxy-D-xylulose-5-phosphate, and 3-OH-C6-HSL. The positive correlation of ruminal 3-OH-C6-HSL with both blood selenium and these enriched taxa suggests a potential link between microbial activity and host selenium status. These findings indicate that HMSeBA supplementation improves the antioxidant and immune status of dairy cows, accompanied by exploratory, hypothesis-generating shifts in the ruminal microbiome and metabolome. Collectively, these findings highlight HMSeBA as a promising nutritional strategy to produce selenium-enriched milk while safeguarding udder health.

## 1. Introduction

Selenium (Se) is an essential trace element for animals, and it plays a critical role in multiple physiological processes, including growth, metabolism, immune function, and reproduction. Selenoproteins serve as the principal functional mediators of Se, with selenocysteine (the 21st amino acid in the genetic code) occupying their catalytic sites [1]. Selenocysteine is indispensable for key biological functions such as antioxidant defense, immunomodulation, and thyroid hormone metabolism. For high-yielding dairy cows, an optimal Se status acts as a safeguard for mammary tissue health and systemic oxidative stability. Conversely, clinical Se deficiencies frequently impair the cow’s defenses against mastitis, resulting in elevated milk somatic cell counts (SCC) [2]. Given that dietary Se levels are profoundly dictated by geographic soil traits, strategic Se supplementation has become a necessity in modern dairy operations.

Dietary Se supplements generally fall into inorganic (sodium selenite) and organic forms (Se-enriched yeast and selenomethionine). Extensive research has confirmed that organic Se is effective in improving the nutritional status of dairy cows [3,4,5]. Compared with organic selenium, inorganic formulations exhibit higher systemic toxicity, poorer bioavailability, and distinct pro-oxidant effects that can trigger cellular oxidative damage [6], driving a growing interest in organic alternatives. Given the narrow margin between dietary selenium deficiency and toxicity, selenium supplementation in dairy cattle diets is subject to strict regulatory oversight in major livestock-producing regions. To minimize the risk of animal toxicity and environmental contamination, selenium inclusion in livestock diets is strictly regulated by global governance bodies. For instance, the United States Food and Drug Administration (FDA) enforces a maximum supplemental selenium limit of 0.3 mg/kg on a dry matter basis for ruminant rations [7], while the European Food Safety Authority (EFSA) mandates a maximum ceiling of 0.5 mg/kg for total selenium content in complete animal feed [8]. These stringent regulatory limits underscore the critical necessity of utilizing alternative selenium sources with superior bioavailability, which can maximize physiological efficacy and antioxidant defense within safe, legally permissible levels. Hydroxy-selenomethionine (HMSeBA), a hydroxy analog of selenomethionine, features high chemical purity and superior bioavailability [9]. The enhanced ruminal stability of HMSeBA relative to other organic selenium forms is attributable to its unique alpha-hydroxy acid structure. Unlike selenomethionine, which possesses an alpha-amino group (–NH_2_) susceptible to microbial transamination and deamination in the rumen, HMSeBA carries a hydroxyl group (–OH) at the alpha carbon position [10]. This structural difference renders HMSeBA resistant to the amino acid-catabolizing enzyme systems of rumen microorganisms, allowing it to traverse the rumen environment largely intact and be absorbed as a whole molecule in the small intestine, thereby contributing to its superior bioavailability compared with other selenium sources [11]. Specifically, HMSeBA is converted in vivo primarily into selenocysteine (SeCys) after absorption in the small intestine [12], which is then directly incorporated into the active sites of selenoproteins like glutathione peroxidase (GSH-Px) to fortify antioxidant defenses [13]. Notably, rather than directly serving as a primary methionine source due to the minimal total dosage supplied (on the milligram scale daily relative to the gram-scale methionine requirements of high-yielding cows [14], the true value of HMSeBA lies in its efficient Se delivery via this pathway. Its advantages as a feed additive for monogastric animals (pigs and poultry) have been well studied [15,16,17], but research on ruminants, particularly dairy cows, remains limited and largely confined to conventional nutritional endpoints. Nevertheless, preliminary studies have shown that HMSeBA supplementation improves the lactation performance, blood parameters, antioxidant capacity, and the Se transfer efficiency of early lactating dairy cows [18]. Crucially, under severe physiological insults such as heat stress, HMSeBA significantly enhances the antioxidant and immune capacity of dairy cows without markedly affecting the milk composition, thereby mitigating heat stress-induced damage to the mammary glands [19]. In line with these benefits, supplementation during heat stress has also been shown to significantly increase Se concentrations in both blood and milk [20], providing direct biochemical evidence for its enhanced bioavailability and efficient translocation across the blood–milk barrier.

Because the rumen operates as the central hub for microbial fermentation, nutrient metabolism, and Se biotransformation in ruminants, the composition and functional capacity of the rumen microbial community critically influence host metabolic homeostasis and health [21]. Organic Se plays an important regulatory role in rumen microbial activity, optimizing rumen fermentation patterns and ultimately promoting nutrient digestibility and utilization [22]. However, while preliminary studies have explored the effects of HMSeBA on dairy cow production, research in the field of dairy cow metabolomics remains limited. Consequently, the molecular pathways and redox-regulatory cascades governed by this organic selenium source within the rumen environment are still undefined. This limited molecular insight restricts our understanding of how localized microbial alterations guide the host’s systemic antioxidant and immune responses.

Therefore, this study employed a multi-omics integrative approach—combining 16S rRNA gene sequencing and untargeted metabolomics—to systematically characterize the effects of HMSeBA on lactation performance, rumen microbiota composition, and ruminal metabolic profiles in dairy cows. The findings are intended to provide a mechanistic basis for the application of HMSeBA in dairy nutrition, and to advance current understanding of the redox-regulatory and immunomodulatory mechanisms associated with organic selenium at the rumen–host interface.

## 2. Materials and Methods

### 2.1. Ethics Statement

All animal procedures were reviewed and approved by the Laboratory Animals Welfare and Ethics Committee of Nanjing Agricultural University (Audit Number: NJAULLSC2024109; Approval date: 15 November 2024).

### 2.2. Animals and Experimental Design

The trial was conducted between December 2024 and March 2025 at a dairy farm affiliated with Nanjing Weigang Dairy Co., Ltd. (Nanjing, China). The Hydroxy-selenomethionine (HMSeBA, Selisseo^®^ 2% Se) was purchased from Adisseo Life Science Co., Ltd. (Shanghai, China). The supplement contains 2.0% total selenium in the form of 2-hydroxy-4-(methylseleno)butanoic acid. Forty Holstein dairy cows (251.65 ± 9.70 d in milk, 33.5 ± 2.01 kg of milk/d, 1.25 ± 0.10 parity) were selected based on similar age, parity, and milk production, and randomly divided into two groups, a control group (CG) and an experimental group (EG) (*n* = 20 per group). These characteristics indicate that all enrolled animals were in mid-to-late lactation at the time of study initiation. At the initiation of the study, the pregnancy rates were 50.0% (10/20) for EG and 45.0% (9/20) for CG. For the pregnant cows, the average days of gestation were 104.2 ± 26.5 d in the EG and 102.9 ± 26.5 d in the CG, showing no significant difference between treatments (*p* > 0.05). The trial period lasted for 105 days, consisting of a 7-day pre-feeding period and a 98-day main trial period. Cows in the CG were fed a basal dairy cow diet (Table 1) containing 0.32 mg/kg Se (DM basis), which satisfied the recommended selenium requirement for lactating dairy cows as specified by NASEM [7]. Cows in the EG received the same basal diet supplemented with HMSeBA, resulting in a total dietary Se concentration of 0.64 mg/kg DM. This supplemental level was selected based on previous studies [11,23], which demonstrated that supranutritional Se supplementation in the range of 0.60–0.90 mg/kg DM using highly bioavailable organic selenium sources significantly enhanced antioxidant capacity, immune function, and milk quality in dairy cows without inducing toxic effects. The experimental cows were housed in a free-stall barn and fed a total mixed ration (TMR) three times daily. They were milked three times daily and had ad libitum access to food and water.

### 2.3. Milk Samples Collection and Analysis

The daily milk yield was recorded throughout the trial, and milk production efficiency (MPE) was calculated as milk yield (kg/d) divided by dry matter intake (DMI, kg/d). According to the Dairy Herd Improvement (DHI) protocol, composite milk samples (4:3:3, morning:midday:evening) were collected biweekly for four months. One preserved aliquot was analyzed for fat, protein, lactose, milk urea nitrogen, and SCC using a DHI system (Bentley NexGen FCM-FTS, Chaska, MN, USA). The other was stored at −20 °C for Se analysis, performed according to the National Standard of the People’s Republic of China: National Food Safety Standard-Determination of Multi-elements in Foods (Standards Press of China, Beijing; GB 5009.268-2025) [24].

### 2.4. Blood Sample Collection and Testing

Blood was drawn from the tail vein at the end of the pre-feeding period and during the trial. Serum, separated after clotting and centrifugation (3500 r/min, 10 min) (Centrifuge model: DLAB Scientific Co., Ltd., Beijing, China; #DM0412), was stored at −80 °C. The serum concentrations of the total protein (TP), albumin (ALB), malondialdehyde (MDA), superoxide dismutase (SOD), and glutathione peroxidase (GSH-Px) were measured using a microplate reader (Spark, TECAN, Männedorf, Switzerland) and commercial assay kits (Nanjing Jiancheng Bioengineering Institute Co., Ltd., Nanjing, China). Immunoglobulins A, G, and M (IgA, IgG, and IgM, respectively) were quantified using a commercial enzyme-linked immunosorbent assay (Nanjing MALLBIO Biological Technology Co., Ltd., Nanjing, China). Blood Se levels were analyzed according to the National Standard of the People’s Republic of China: National Food Safety Standard—Determination of Multiple Elements in Foods (Standards Press of China, Beijing; GB 5009.268-2025) [24].

### 2.5. Rumen Fermentation Parameters

For rumen fluid sampling, a representative subset of 10 cows (5 cows per group) was selected from the total cohort of 40 cows. To minimize compounding biological noise and avoid confounding individual physiological outliers in downstream metabolomics, cows with milk yield and malondialdehyde (MDA) concentrations around the group median were selected, resulting in the exclusion of 30 cows from rumen sampling. To verify that the sampled subset accurately represented the entire experimental population, key background and physiological characteristics were statistically compared. As documented in Supplementary Table S1, the selected cows demonstrated no significant deviations from the overall study population (*p* > 0.05) in terms of parity, days in milk (DIM), selenium status, and SCC, validating the absence of selection bias. On the final trial day, rumen fluid was collected from five selected cows per group (*n* = 5) via an oral stomach tube 2–4 h before the morning feed. The pH of the rumen fluid was immediately measured using a portable pH meter (0.01 pH resolution; Lichen Instrument Co., Ltd., Shanghai, China). The filtered rumen fluid was then divided into aliquots, where samples for volatile fatty acid (VFA) analysis were acidified with 25% metaphosphoric acid and analyzed via a Shimadzu GC-14B gas chromatograph (Shimadzu Corp., Kyoto, Japan). Concentrations of ammonia nitrogen (NH_3_-N) [25] and microbial crude protein (MCP) [26] were determined using spectrophotometric colorimetric assays. The remaining ruminal aliquots were flash-frozen in liquid nitrogen and stored at −80 °C prior to 16S rRNA sequencing and untargeted metabolomics.

### 2.6. 16S rRNA Microbial Sequencing

The total microbial genomic DNA was extracted using the E.Z.N.A.^®^ soil DNA Kit (Omega Bio-tek, Norcross, GA, USA) according to the manufacturer’s instructions. The quality and concentration of the DNA were determined using 1.0% agarose gel electrophoresis and a NanoDrop2000 spectrophotometer (Thermo Scientific, Waltham, MA, USA). To monitor potential background contamination from reagents or the laboratory environment, DNA extraction blank controls (negative controls using sterile water) were processed in parallel with the ruminal fluid samples. The hypervariable region V3-V4 of the bacterial 16S rRNA gene was amplified with primer pairs 338F (5′-ACTCCTACGGGAGGCAGCAG-3′)/806R (5′-GGACTACHVGGGTWTCTAAT-3′) by T100 Thermal Cycler PCR thermocycler (BIO-RAD, Hercules, CA, USA) [27]. The PCR mixture (20 µL) contained Fast Pfu buffer, dNTPs, primers, Fast Pfu polymerase, and template DNA. Amplification was performed under the following conditions: 95 °C for 3 min; 27 cycles of 95 °C for 30 s, 55 °C for 30 s, and 72 °C for 45 s; and a final extension at 72 °C for 10 min. The PCR products were purified and quantified by sequencing on an Illumina Nextseq2000 platform at Majorbio Bioinformatics Technology Co., Ltd. (Shanghai, China). Raw FASTQ files were de-multiplexed using an in-house Perl script, and then quality-filtered by fastp version 0.19.6 [28] and merged by FLASH version 1.2.7 [29]. After quality filtering and merging of paired-end reads, a total of 609,923 high-quality sequences were obtained across all 10 samples. The sequencing depth was robust, with an average of 60,992 sequences per sample. This depth of coverage is sufficient to capture the taxonomic diversity and community structure of the rumen microbiota in this study. The optimized sequences were then clustered into operational taxonomic units (OTUs) using UPARSE 7.1 with 97% sequence similarity level [30]. The taxonomy of each OTU representative sequence was analyzed by RDP Classifier version 2.2 against the 16S rRNA gene database using a confidence threshold of 0.7 [31]. Using the Vegan v2.5-3 package, the PERMANOVA test was employed to assess the percentage of variation explained by the treatment, along with its statistical significance. Alpha-diversity metrics were calculated using Mothur v1.30.1 [32]. A linear discriminant analysis (LDA) effect size (LEfSe) was performed to identify significantly abundant taxa (phylum to genera) of bacteria among the different groups (LDA score > 2, *p* < 0.05) [33].

### 2.7. Rumen Untargeted Metabolomics Analysis

A rumen metabolomic analysis was performed using LC-MS on a Thermo UHPLC-Exploris480 system equipped (Thermo Fisher Scientific, Waltham, MA, USA) with an ACQUITY HSS T3 column (100 mm × 2.1 mm i.d., 1.8 μm; Waters, Milford, MA, USA) at Majorbio Bio-Pharm Technology Co. Ltd. (Shanghai, China). Raw LC-MS data were preprocessed using Progenesis QI software (Waters Corporation, Milford, CT, USA). The metabolites were identified by searching the HMDB (http://www.hmdb.ca/), Metlin (https://metlin.scripps.edu/), and Majorbio internal databases. Metabolite identification was performed using MS/MS spectral matching at two confidence levels: level B(i), representing matching against an in-house experimental MS/MS spectral library with high annotation confidence, and level B(ii), representing matching against computer-simulated theoretical MS/MS spectra in public databases. Both levels correspond to MSI Level 2 putative annotation [34]. Biological interpretation and pathway analysis were restricted to metabolites annotated at the B(i) or B(ii) level, with B(ii) annotations interpreted with additional caution given their reliance on theoretical spectral prediction. To monitor analytical stability, QC samples were prepared by pooling all samples and were injected into every 5–15 samples throughout the run. Metabolites with a relative standard deviation (RSD) > 30% in QC samples were excluded from downstream analysis to ensure data reliability. Then, the R ropls (Version 1.6.2) package was used to perform principal component analysis (PCA) and orthogonal least partial squares discriminant analysis (OPLS-DA), alongside a 7-cycle interactive validation to evaluate model stability. Differential metabolites between the CGs and EGs were initially screened based on a variable importance in projection (VIP) score > 1.0 derived from the OPLS-DA model, combined with a univariate Student’s *t*-test *p*-value < 0.05. To account for the large number of variables examined and to control the false discovery rate (FDR) arising from multiple comparisons, *p*-values were further adjusted using the Benjamini–Hochberg (BH) procedure [35]. Metabolites meeting the criteria of VIP > 1.0, *p* < 0.05, and FDR-adjusted *p* < 0.1 were retained as high-confidence differential metabolites. This FDR threshold was selected in accordance with conventional practice in untargeted metabolomics studies, given the inherently higher false-positive risk associated with the large number of simultaneously tested metabolic features [36]. Differential metabolites between the two groups were mapped to their biochemical pathways through metabolic enrichment and pathway analysis based on the KEGG database (http://www.genome.jp/kegg/ (accessed on 12 June 2025)).

### 2.8. Statistical Analysis

All data were initially processed using Microsoft Excel 2021 and subsequently analyzed using IBM SPSS Statistics (version 26.0; IBM Corp., Armonk, NY, USA). Data on lactation performance and milk Se were analyzed using linear mixed-effects models (LMMs) with repeated measures. In the model structure, Se treatment (CG or EG), time (0, 15, 30, 45, 60, 75, 90, 105 d), and their interaction (Se × time) were specified as fixed effects, while individual cow was included as a random effect to account for the correlation among repeated measurements within the same animal. To determine the optimal within-subject correlation structure, several covariance matrices were evaluated using the Akaike Information Criterion (AIC), and the unstructured (UN) covariance matrix was selected as it yielded the lowest AIC value. Parameters were estimated using the restricted maximum likelihood (REML) method, and denominator degrees of freedom were approximated using the Kenward–Roger method. Effects were considered statistically significant at *p* < 0.05. Data regarding serum parameters, blood Se, and ruminal fermentation parameters were analyzed using an independent samples *t*-test, with ruminal variables presented as mean values alongside the pooled standard error of the mean (SEM). Statistical significance was set at *p* < 0.05.

Rumen microbiome data were analyzed using the Mann–Whitney U-test, and significance was determined using false discovery rate (FDR)-adjusted *p*-values (*p* < 0.05). Metabolomic data were analyzed using multivariate statistical approaches, and differential metabolites were identified based on VIP > 1.0, *p* < 0.05, and FDR-adjusted *p* < 0.1. Figures were created using GraphPad Prism version 10.1.2 (GraphPad Software, San Diego, CA, USA). Additionally, Spearman’s rank correlation analysis was performed to evaluate relationships among the rumen microbiota, milk Se, blood Se, and significantly altered rumen metabolites.

## 3. Results

### 3.1. Feed Intake and Lactation Performance

**Figure 1:**
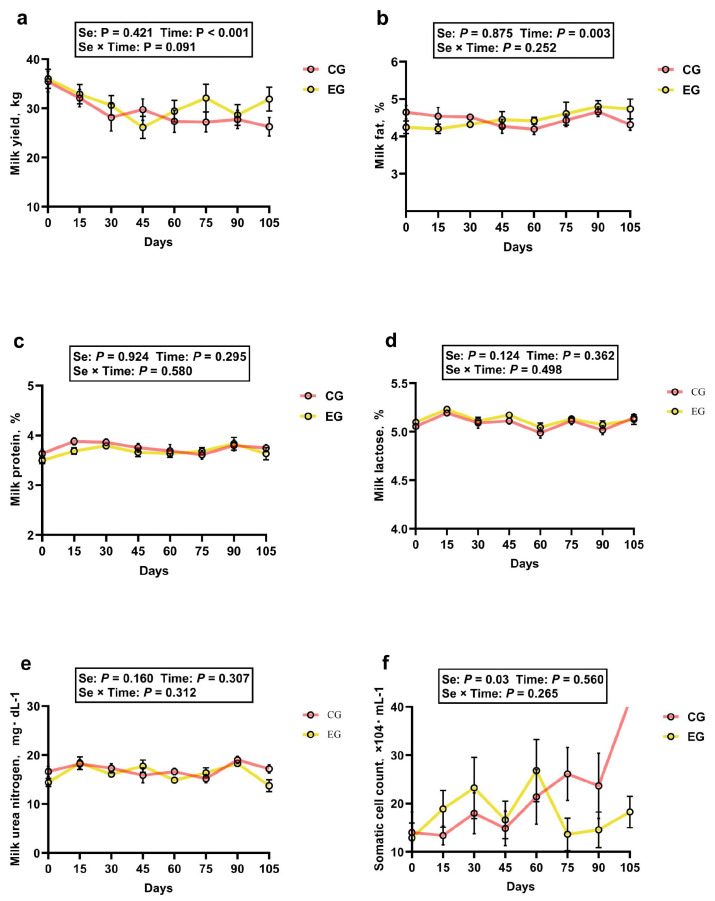
Effects of HMSeBA supplementation on lactation performance in dairy cows. (**a**) Milk yield; (**b**) milk fat; (**c**) milk protein; (**d**) milk lactose; (**e**) milk urea nitrogen; (**f**) somatic cell count.

Throughout the 105-day trial period, no significant difference in DMI was observed between the groups, although a downward trend was noted in the EG (*p* = 0.075); similarly, MPE exhibited no significant difference between treatments (*p* > 0.05) (shown in Table 2). The lactation performance of the cows in both groups over time is shown in Figure 1. No differences were found (*p* > 0.05) in milk yield, milk fat, milk protein, milk lactose, or milk urea nitrogen between treatments, except for the SCC in milk, which was lower in the EG than in the CG (*p* < 0.05). However, a time effect was observed for milk yield and fat content (*p* < 0.05) with fluctuations throughout the trial in both groups. In addition, no interactions between Se and time were observed for the lactation performance (*p* > 0.05).

### 3.2. Blood and Milk Concentrations of Selenium

**Figure 2:**
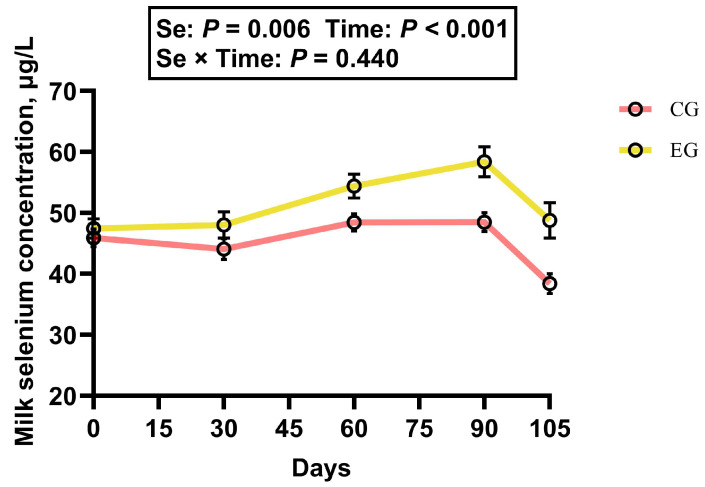
Effects of HMSeBA supplementation on milk selenium concentration in dairy cows.

HMSeBA supplementation significantly increased the Se concentrations in both the milk and blood of dairy cows (shown in Figure 2 and Table 3). The Se content of milk was significantly higher in the EG than in the CG (*p* < 0.05). A significant effect of time was also observed, with milk Se levels varying throughout the experimental period in both groups (*p* < 0.05). However, no significant interaction was detected between Se and time (*p* > 0.05). Furthermore, no difference in blood Se concentrations was detected between the CG and EG before the trial (*p* > 0.05), but following the trial, a significant increase in the blood Se concentration was observed in the EG compared to that in the CG (*p* < 0.05).

### 3.3. Serum Biochemical, Immune, and Antioxidative Parameters

**Figure 3:**
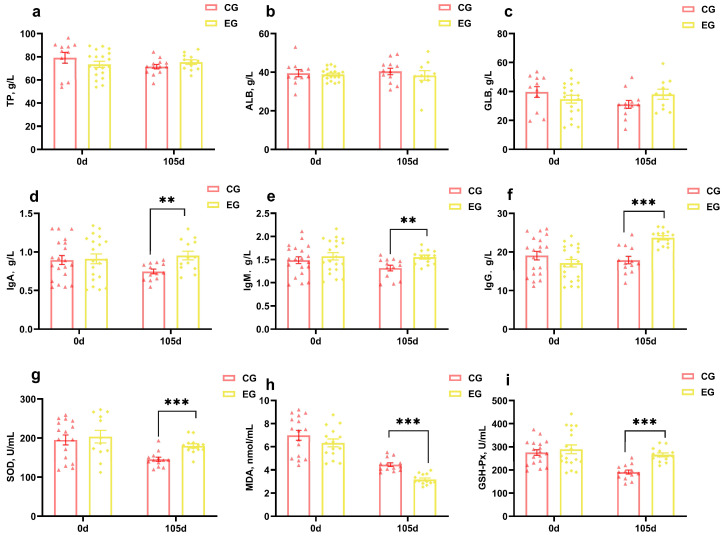
Effects of HMSeBA on serum biochemical, immune, and antioxidative parameters in dairy cows. (**a**) Total protein, (**b**) albumin, (**c**) globulin, (**d**) immunoglobulin A, (**e**) immunoglobulin M, (**f**) immunoglobulin G, (**g**) superoxide dismutase, (**h**) malondialdehyde, (**i**) glutathione peroxidase. ** *p* < 0.01, *** *p* < 0.001. Triangles and diamonds represent individual data points for the control group (CG) and experimental group (EG), respectively.

The effects of HMSeBA supplementation on the serum biochemical, immune, and antioxidative parameters in dairy cows are presented in Figure 3. Before the trial, there were no significant differences in the serum biochemical, immune, or antioxidative parameters between the two groups (*p* > 0.05). After the trial, compared with the CG, the EG showed significantly increased serum concentrations of immune parameters (immunoglobulin A, immunoglobulin M, and immunoglobulin G) and antioxidative parameters (superoxide dismutase and glutathione peroxidase) (*p* < 0.05), along with a significantly decreased level of malondialdehyde (*p* < 0.05). No significant differences were observed in the serum biochemical parameters between the two groups (*p* > 0.05).

### 3.4. Rumen Fermentation Parameters

The effects of dietary HMSeBA supplementation on ruminal fermentation characteristics are presented in Table 4. HMSeBA supplementation significantly increased the concentration of butyrate in rumen fluid compared with the CG (*p* < 0.05). Numerical increases were also observed in the concentrations of acetate, total volatile fatty acids, microbial crude protein (MCP), and isovaleric acid in the EG, though these differences did not reach statistical significance (*p* = 0.052, 0.067, 0.058, and 0.077, respectively). Dietary HMSeBA supplementation did not significantly affect ruminal pH or the concentrations of NH3-N, propionate, or valeric acid (*p* > 0.05).

### 3.5. Rumen Microbiota

**Figure 4:**
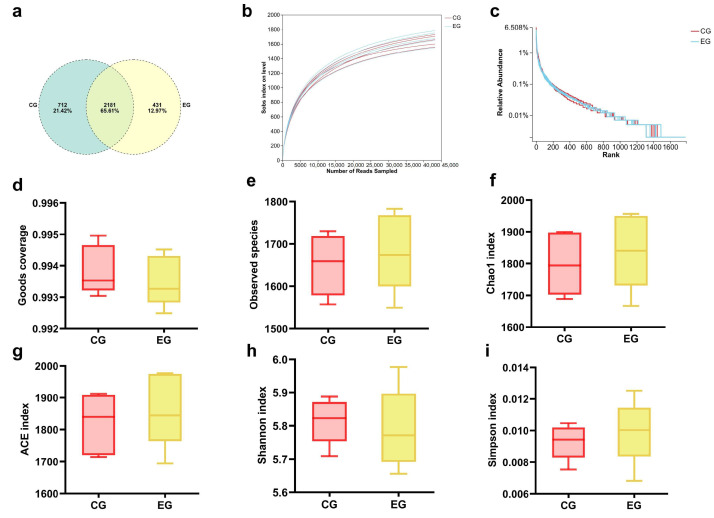
Effects of HMSeBA supplementation on rumen microbiota in dairy cows. (**a**) Venn diagram analysis of the ruminal microbial OTUs, (**b**) rarefaction curve of alpha diversity, (**c**) rank abundance curve, (**d**) goods coverage, (**e**) observed species, (**f**) Chao1 index, (**g**) ACE index, (**h**) Shannon index, (**i**) Simpson index.

**Figure 5:**
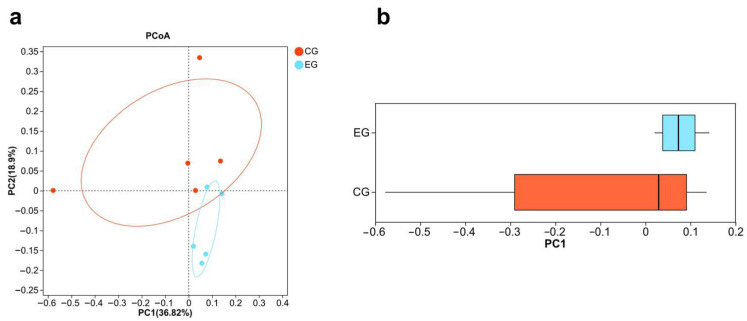
Effects of HMSeBA supplementation on rumen microbial beta-diversity in dairy cows. (**a**) PCoA plot based on the Bray–Curtis distance matrix at the OTU level. Red and blue ovals represent the 95% confidence ellipses for the CG and EG groups, respectively, and (**b**) box-and-whisker plot illustrating the distribution of the first principal coordinate (PC1) scores.

To comprehensively assess the macro-structural variations and sequencing depth of the ruminal microbiota following HMSeBA supplementation, a joint analysis of operational taxonomic unit (OTU) distribution and alpha diversity indices was conducted. As illustrated in Figure 4a, a shared core of 2181 OTUs was common to both groups, accounting for 65.61% of the total identified microbial population. Concurrently, a total of 712 and 431 OTUs were uniquely clustered within the CG and the EG, respectively. To confirm sampling proficiency, the sample-based rarefaction curves of microbial species richness and the rank-abundance curves were evaluated (Figure 4b,c). Furthermore, to demonstrate the variations in beta diversity among the groups, a principal coordinate analysis (PCoA) paired with a PC1 score distribution plot was performed (Figure 5).

**Figure 6:**
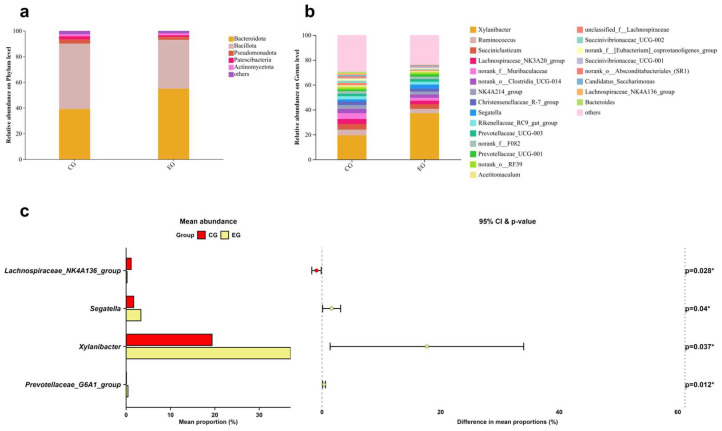
Effects of HMSeBA supplementation on ruminal microbial community composition and differential taxa analysis in dairy cows. (**a**) Relative abundance of bacterial phylum in the rumen, (**b**) relative abundance of bacterial genera in the rumen, (**c**) relative abundance of differential microorganisms at the genus level in dairy cows. Red and yellow bars represent the CG and EG groups, respectively. Dots in the right panel represent the difference in mean proportions between groups, with horizontal lines indicating 95% confidence intervals. * *p* < 0.05.

Taxonomic profiling of the ruminal microbiota was characterized at both phylum and genus tiers to evaluate the community shifts following HMSeBA supplementation. At the phylum level, *Bacteroidota* and *Bacillota* represented the two most predominant bacterial phyla, collectively encompassing over 90% of the total sequences (Figure 6a). At the genus level, 241 microbial taxa were detected in the rumen fluid samples, and the top 50 bacterial genera in the cow rumen are shown in the bar chart in Figure 6b. To investigate the bacterial community structure at depth, we defined dominant genera as those with an average relative abundance exceeding 1%. According to this criterion, the dominant genera in the CG, listed in descending order of mean relative abundance, were *Xylanibacter* (19.38%), *Ruminococcus* (4.63%), *Succiniclasticum* (4.6%), *Lachnospiraceae*_NK3A20_group (4.41%), *Christensenellaceae*_R-7_group (2.81%), *Segatella* (2.57%), and *Rikenellaceae*_RC9_gut_group (2.02%). In the EG, the dominant genera, listed in descending order, were *Xylanibacter* (37.07%), *Succiniclasticum* (3.75%), *Ruminococcus* (3.58%), *Segatella* (3.34%), *Lachnospiraceae*_NK3A20_group (2.82%), *Rikenellaceae*_RC9_gut_group (2.31%), and *Christensenellaceae*_R-7_group (2.26%). Four genera showed significant differences (Figure 6c). Among them, the relative abundances of *Prevotellaceae*_Ga6A1_group, *Xylanibacter*, and *Segatella* were significantly higher in the EG than in the CG, whereas the relative abundance of *Lachnospiraceae*_NK4A136_group was significantly lower (*p* < 0.05).

### 3.6. Rumen Metabolome

**Figure 7:**
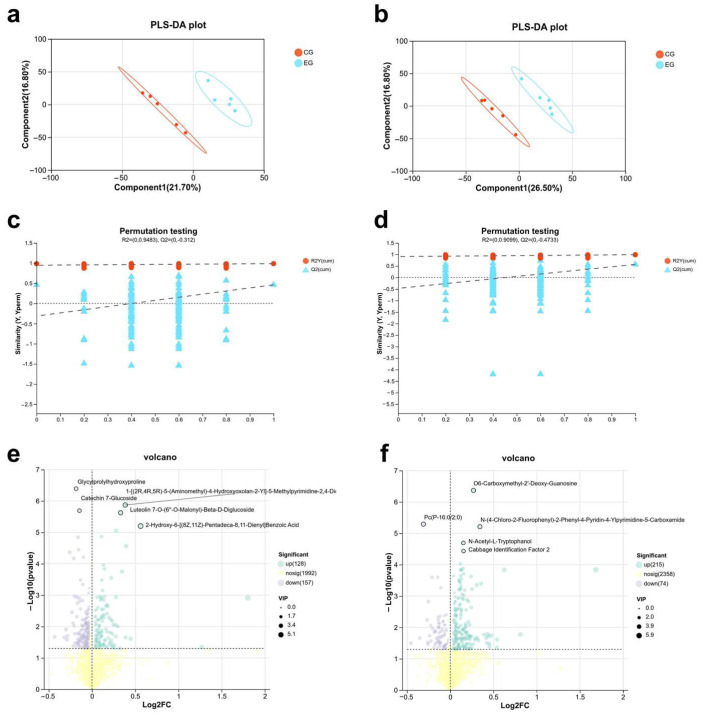
Effects of HMSeBA supplementation on multivariate statistical analysis of the rumen fluid metabolome. (**a**) PLS-DA score plot in the positive ion mode (R^2^ X (cum) = 0.385, R^2^Y (cum) = 0.987, and Q^2^ (cum) = 0.463); (**b**) PLS-DA score plot in the negative ion mode (R^2^ X (cum) = 0.432, R^2^Y (cum) = 0.991, and Q^2^ (cum) = 0.569). Ellipses represent the 95% confidence intervals for the CG (red) and EG (blue) groups; (**c**) permutation test for the PLS-DA model in the positive ion mode; (**d**) permutation test for the PLS-DA model in the negative ion mode. The dashed line represents the regression trend of permuted Q2 values; the dotted line indicates y = 0.; (**e**) volcano plot of rumen fluid differential metabolites in the positive ion mode; (**f**) volcano plot of rumen fluid differential metabolites in the negative ion mode.

The partial least squares-discriminant analysis (PLS-DA) score plots revealed a distinct separation and clear clustering between the CG and EG along the first principal component, both in positive and negative ionization modes (Figure 7a,b). This profound separation indicated that HMSeBA supplementation substantially altered the rumen metabolic profile of dairy cows. To evaluate model validity and rule out overfitting, 200 times permutation tests were performed. In the positive and negative ionization modes, the Q^2^ regression lines yielded negative *Y*-axis intercepts of −0.312 (Figure 7c) and −0.4733 (Figure 7d), respectively. These negative intercepts indicated the validity and robustness, suggesting that the metabolic separation was driven by underlying biological treatment effects rather than statistical artifacts or random chance. To identify differentially abundant metabolites, a combined criterion was applied: variable importance in projection (VIP) > 1, along with a *p*-value of < 0.05. Based on these thresholds, the volcano plots illustrated numerous significantly altered features (Figure 7e,f). Following rigorous chemical structure identification, a total of 574 key differentially abundant metabolites were confirmed. In the positive ion mode, 128 of these identified metabolites were significantly upregulated, and 157 were downregulated, whereas in the negative ion mode, 215 were significantly upregulated and 74 were downregulated. The high reliability and clear contrast demonstrated by these screened biomarkers further validated their efficacy in distinguishing the metabolic signatures between the CGs and EGs.

**Figure 8:**
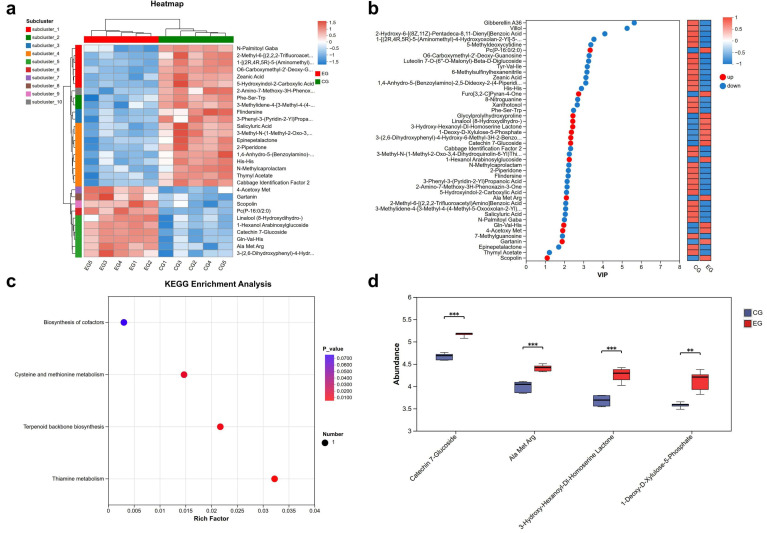
Effects of HMSeBA supplementation on differential metabolites and metabolic pathways in rumen fluid. (**a**) Hierarchical clustering heat map of differential metabolites, full metabolite names corresponding to the abbreviated labels are provided in Supplementary Table S3, (**b**) VIP bar plot displaying metabolites, (**c**) KEGG enrichment analysis, (**d**) relative abundances of key metabolites. ** *p* < 0.01, *** *p* < 0.001.

Among 2618 metabolites initially identified in the ruminal fluid samples, 1411 were retained after applying stringent quality-filtering criteria. Subsequent statistical screening based on OPLS-DA and univariate criteria (VIP > 1.0 and *p* < 0.05) yielded 574 differential candidates. To control the false discovery rate (FDR) arising from multiple comparisons, *p*-values were adjusted using the Benjamini–Hochberg procedure. After applying the stringent FDR threshold (FDR-adjusted *p* < 0.1) and excluding metabolites with biologically implausible annotations in the context of bovine rumen fluid, a total of 44 high-confidence differential metabolites were ultimately identified between the EGs and CGs (Table S3). Among these final candidates, 14 were upregulated, and 30 were downregulated in the EG. A complete summary of this metabolite filtering workflow is provided in Supplementary Table S2. As shown in Figure 8a, CG and EG clustered into two distinct groups, indicating significant metabolic differences between the two groups. The differential metabolites effectively distinguished rumen fluid samples from the different groups, and high reproducibility was observed within each group. These results confirm the reliability of the PLS-DA model in discriminating between groups and indicate that dietary supplementation with HMSeBA substantially alters the rumen fluid metabolome in dairy cows. As illustrated in Figure 8b, the top differential metabolites were ranked according to their VIP values, reflecting their relative contribution to the separation of the two experimental groups. Among these identified compounds, a clear regulatory shift was observed, with 14 metabolites significantly upregulated and 30 metabolites downregulated in response to HMSeBA supplementation. KEGG pathway enrichment analysis of the 44 differential metabolites identified 1-Deoxy-D-Xylulose-5-Phosphate (DXP) as the sole compound mapped to significantly enriched pathways, with enrichment observed in thiamine metabolism, terpenoid backbone biosynthesis, biosynthesis of cofactors, and cysteine and methionine metabolism (Figure 8c). Given that pathway enrichment was driven by a single metabolite, these results are presented as exploratory observations. Four representative differential metabolites with the highest VIP scores were selected for further visualization (Figure 8d). Among these, Catechin 7-Glucoside, Ala-Met-Arg, 3-Hydroxy-Hexanoyl-DL-Homoserine Lactone (3-OH-C6-HSL), and 1-Deoxy-D-Xylulose-5-Phosphate (DXP) were significantly elevated in the EG (*p* < 0.001, *p* < 0.001, and *p* < 0.01, respectively).

**Figure 9:**
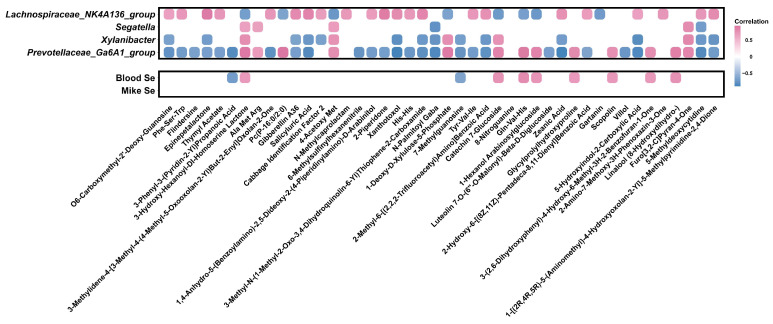
Correlation analysis between rumen metabolites with dominant bacterial genera and Se status in dairy cows. Red represents a positive correlation, blue represents a negative correlation, and the darker the color, the higher the correlation.

To further explore the interactions among rumen microbiota, metabolites, and Se status in dairy cows, an integrated multi-omics correlation analysis was performed (shown in Figure 9). 3-OH-C6-HSL exhibited significant positive correlations with blood selenium concentration (r = 0.68, *p* = 0.029), as well as the relative abundances of *Prevotellaceae*_Ga6A1_group (r = 0.92, *p* = 0.0002), *Xylanibacter* (r = 0.77, *p* = 0.009), and *Segatella* (r = 0.65, *p* = 0.043). Conversely, it showed a significant negative correlation with the relative abundance of *Lachnospiraceae*_NK3A20_group (r = −0.68, *p* = 0.030).

## 4. Discussion

HMSeBA supplementation had no detrimental effects on milk yield or regular milk components, but it significantly reduced the milk SCC. This finding aligns with previous studies demonstrating that adding organic Se to mineral salt blocks exerts no significant effects on milk yield, fat, protein, or lactose in dairy cows, while significantly reducing milk SCC [37]. In contrast, alternative research reported that HMSeBA enhances the lactation performance in early-lactation cows [18]. This discrepancy with the findings of Li et al. can be attributed to distinct baseline selenium backgrounds, experimental dosages, and physiological stages. First, our basal diet was Se-adequate (0.32 mg/kg DM), whereas Li et al. evaluated the correction of a severe Se deficiency (0.04 mg/kg DM), a context in which production responses are typically maximized. Second, Li et al. reported a quadratic dose–response where lactation performance and antioxidant indices peaked at 0.14–0.34 mg Se/kg DM but plateaued and declined at 0.54 mg/kg DM. Our dietary Se concentration (0.64 mg/kg DM) exceeds this upper threshold, suggesting it lies beyond the range for driving further production benefits. Third, our cows were in mid-to-late lactation (approximately 250 DIM) and characterized by greater metabolic stability, whereas the early-lactation cows (57 DIM) in Li et al. faced pronounced negative energy balance and intense metabolic demands. Notably, when the baseline Se status of dairy cows already meets the requirements for maintaining normal production, additional Se supplementation rarely triggers further production-enhancing effects [38]. More importantly, he observed pattern of results is more consistent with HMSeBA primarily supporting the host immune-antioxidant defense system, rather than directly accelerating the raw synthesis of milk macromolecule components. High-yielding dairy cows during intense lactation exhibit highly accelerated metabolic rates, fueling an overproduction of reactive oxygen species (ROS) that frequently inflicts oxidative damage on the mammary epithelium [39]. Consequently, these outcomes indicate that under an adequate dietary baseline, the antioxidant efficacy of HMSeBA supplementation may be more closely linked to scavenging excessive ROS accumulation and safeguarding the structural integrity of the blood–milk barrier, which may help maintain mammary epithelial health without introducing further alterations to regular milk macromolecule synthesis.

The biological efficacy of selenium (Se) in fortifying antioxidant defenses and immune function is well-established [40]. In this study, dietary HMSeBA supplementation significantly increased serum IgA, IgM, and IgG levels, while elevating SOD and GSH-Px activities and reducing MDA content. The enhancement in systemic selenium status likely reflects the post-ruminal utilization characteristics of HMSeBA. As a hydroxy analog of selenomethionine, HMSeBA resists ruminal degradation and is absorbed in the small intestine via amino acid transporters, exhibiting superior bioavailability compared to inorganic selenium sources [41]. Following absorption, HMSeBA enters the selenomethionine pool and is subsequently metabolized to selenocysteine (Sec), the biologically active form of selenium [12]. Sec is cotranslationally incorporated, via a unique UGA-recoding mechanism, into the active site of selenoproteins, including glutathione peroxidase [42]. As a key Se-dependent enzyme, GSH-Px reduces lipid hydroperoxides and other peroxides to limit oxidative damage [13], consistent with the diminished MDA concentrations observed in HMSeBA-supplemented cows. These changes indicate enhanced selenium availability for selenoprotein synthesis and function, though selenium speciation and expression were not directly measured. Beyond the GPx pathway, Se-dependent systems such as the thioredoxin (Trx) system [43], may contribute to intracellular redox balance and modulate redox-sensitive inflammatory signaling. This may offer a plausible, though unconfirmed, explanation for the concurrent increases in circulating immunoglobulins and the decline in SCC observed here. However, because Trx activity, NF-κB signaling, and mammary inflammation were not directly assessed, these pathways should be interpreted as logical hypotheses rather than direct evidence.

Volatile fatty acids (VFAs) produced by ruminal microbial fermentation constitute a fundamental energy source for ruminants, accounting for approximately 70% of the metabolizable energy required by dairy cows [44]. In the present study, dietary HMSeBA supplementation significantly increased the concentration of ruminal butyrate, indicating a distinct shift in the fermentation pattern. As a critical energy substrate, butyrate is primarily utilized by the ruminal epithelium and is closely associated with epithelial development and absorptive functions [45]; thus, this elevation likely reflects a biologically meaningful improvement in the localized ruminal environment. Furthermore, butyrate can participate in systemic energy metabolism upon its conversion to β-hydroxybutyrate [46]. Notably, the robust increase in butyrate concentrations observed herein is consistent with prior findings [47]. While the precise mechanisms driving these fermentative changes and the associated shifts in microbial community composition warrant further elucidation, several plausible pathways can be hypothesized based on the chemical traits of HMSeBA. First, the transient presence of the un-bypassed HMSeBA fraction in the ruminal fluid may subtly modulate the local redox microenvironment. This could potentially create conditions more favorable to strictly anaerobic, fiber-degrading taxa—including members of the *Prevotellaceae* family—which are characteristically more susceptible to oxidative damage than facultative anaerobes [48]. Second, the trace amount of organic selenium and methionine analog partitioned into the ruminal fluid phase during HMSeBA transit may provide targeted support for the activity of bacterial selenomethionine-containing enzymes (such as formate dehydrogenase). These enzymes are encoded in the genomes of several *Prevotellaceae* species and may contribute to their metabolic competitiveness within the ruminal community [49]. These proposed mechanisms are offered as exploratory hypotheses consistent with the established ruminal bypass properties of HMSeBA and the observed microbiome data, and require targeted experimental validation. The numerical increases in acetate, total VFA concentrations, and microbial crude protein (MCP) further support the hypothesis that HMSeBA can, to some extent, enhance ruminal fermentation efficiency [47]. However, given that the present study did not directly evaluate ruminal epithelial responses, redox status, or the causal relationships between the microbiota and metabolites, caution should be exercised when interpreting the underlying mechanisms driving these fermentative shifts.

Given the limited understanding of how HMSeBA supplementation may relate to ruminal microecology, this study integrated 16S rRNA gene sequencing with untargeted metabolomics. This multi-omics approach enabled a systematic investigation of the effects of HMSeBA on the composition and metabolic functions of rumen microbiota, thereby comprehensively evaluating its application potential in lactating dairy cows.

At the genus level, *Prevotellaceae*_Ga6A1_group, *Xylanibacter*, and *Segatella* were significantly enriched in the rumen of dairy cows supplemented with HMSeBA. These bacterial taxa are commonly associated with the utilization of non-cellulosic carbohydrates and plant polysaccharides [50]; therefore, their enrichment potentially indicates a shift in the microbial community toward a more active state of carbohydrate fermentation [51]. This interpretation aligns with the increased ruminal butyrate concentration and the numerical upward trend of acetate observed in the current study. Previous studies have likewise demonstrated that supplementation with various methionine analogs or selenium-containing amino acid derivatives can alter the abundance of *Prevotellaceae*-related clades in ruminants, such as goats [52,53]. This cross-species and cross-compound similarity raises the possibility that compounds possessing a methionine-like organic backbone may be associated with a broad, conserved influence on the ruminal microbial ecosystem. In contrast, the relative abundance of *Lachnospiraceae*_NK4A136_group was significantly reduced following HMSeBA administration. However, given the functional heterogeneity within ruminal bacterial genera and the inherent taxonomic resolution limitations of 16S rRNA sequencing [50], these associations should be interpreted with caution rather than as definitive evidence of specific metabolic activities. More broadly, the microbial alterations observed herein indicate that HMSeBA may modulate ruminal fermentation pathways to a certain degree, whereas the enhancement of host antioxidant status is more plausibly explained by the heightened utilization of selenium at the systemic level. Because independent controls for the organic backbone and the selenium moiety were not established in this trial, and direct measurements of metabolite transport or mammary signaling pathways were not performed, the precise relative contributions of these distinct pathways remain to be further elucidated.

Beyond the observed shifts in microbial community composition, parallel changes were detected in several ruminal metabolites, none of which appear to be direct metabolic derivatives of HMSeBA itself. Rather, these compounds are more consistent with dietary-derived compounds subjected to microbial biotransformation or with metabolic intermediates synthesized by the rumen microbiota. Of the 44 differential metabolites identified, 19 were plant-derived secondary metabolites, including flavonoid and coumarin glycosides, terpenoid lactones, and phenolic esters, of which six were elevated in EG cows. Members of the *Prevotellaceae* family, including the enriched genera *Prevotellaceae*_Ga6A1_group, *Xylanibacter*, and *Segatella*, possess exceptionally large and diverse repertoires of glycoside hydrolase (GH) genes, underlying their well-documented capacity to degrade non-cellulosic plant polysaccharides such as hemicellulose, pectin, starch, and oligosaccharides [54]. Given this established enzymatic capacity, the elevated ruminal abundance of catechin 7-glucoside—selected as representative flavonoid glycosides for further visualization—may plausibly reflect altered microbial glycoside-hydrolyzing activity accompanying the observed shifts in these fiber-degrading taxa, rather than a direct effect of HMSeBA.

In addition to their fibrolytic capacity, *Prevotellaceae* species are also recognized for proteolytic activity and peptide utilization [55]. Among six peptide-derived metabolites identified, three were decreased (Phe-Ser-Trp, His-His, and Tyr-Val-Ile) and three were increased (Glycylprolylhydroxyproline, Gln-Val-His, and Ala-Met-Arg) in EG cows, indicating bidirectional changes in the ruminal peptide pool rather than a uniform directional shift. This pattern may reflect a more active cycle of ruminal peptide turnover—encompassing both proteolytic release and microbial peptide utilization—which loosely parallels the numerically higher microbial crude protein (MCP) synthesis observed in EG cows in the present study (*p* = 0.058). However, given that the MCP difference did not reach statistical significance and that peptide identifications were based on database matching without validation using authentic chemical standards, this association should be regarded as a preliminary and exploratory observation.

Further suggesting a potentially more active metabolic state within the microbial community of EG cows, 1-deoxy-D-xylulose-5-phosphate, a bacterial isoprenoid pathway intermediate linked to microbial thiamine biosynthesis [56], and 3-hydroxy-hexanoyl-DL-homoserine lactone, a bacterial quorum-sensing signal positively correlated with the same fiber-degrading genera and with host selenium status, were both elevated in EG cows. Collectively, these findings suggest that HMSeBA supplementation may have altered the ruminal microecological environment, with this shift accompanied by parallel changes in feed-derived flavonoids, peptide pool dynamics, and microbially synthesized metabolic intermediates, rather than reflecting a direct biochemical mechanism of HMSeBA action. Given the exploratory nature of these associations and the limited sample size, these findings should be interpreted as hypothesis-generating and warrant confirmation through targeted analyses in future studies.

Although the present study provides novel insights into the positive effects of HMSeBA on dairy cows, several limitations should be acknowledged. First, the sample size for the multi-omics analysis was relatively small (*n* = 5 per group), which may constrain statistical power and the broad generalizability of the microbiome and metabolome profiles. Consequently, these multi-omics associations remain primarily correlational, and larger validation cohorts are required to confirm these initial findings.

Second, while stringent metabolite annotation criteria (*p* < 0.05, VIP > 1, FDR < 0.1) were applied to minimize false positives, this rigorous filtering reduced the final pool to 44 high-confidence differential metabolites, inevitably restricting the statistical power of downstream pathway enrichment analyses. Furthermore, although metabolite annotation relied on MS/MS spectral matching—corresponding to the Metabolomics Standards Initiative (MSI) Level 2 putative identification—definitive structural confirmation using authentic chemical standards was not performed. Therefore, these individual metabolite identifications and enriched pathways should be interpreted as exploratory, underscoring the need for future targeted metabolomics and validation using authentic reference standards. In particular, B(ii)-level annotations—based on computer-simulated rather than experimentally acquired MS/MS spectra—carry a higher inherent risk of misidentification than B(i)-level annotations, and the specific compound identities of several differential metabolites discussed herein should be regarded as putative until confirmed by authentic reference standards.

Third, the absence of host transcriptomic and targeted proteomic data precludes the definitive verification of exact intracellular signaling cascades and molecular mechanisms within key host tissues, such as the rumen epithelium and mammary gland. Thus, the proposed mechanistic pathways represent highly plausible biological hypotheses rather than definitive causal links. Additionally, the animals enrolled in this study were strictly in mid-to-late lactation (approximately 250 DIM). Because periparturient and early-lactation cows experience substantially greater oxidative stress, negative energy balance, and immune depression, their physiological responses to HMSeBA may differ significantly. Accordingly, these findings should not be broadly extrapolated to other lactation stages, and future investigations during the transition period are highly recommended.

Fourth, this study compared HMSeBA strictly against a basal diet without including alternative selenium sources (e.g., sodium selenite, selenium yeast, or selenomethionine). Thus, while our findings characterize the specific physiological and multi-omics impacts of HMSeBA, they do not demonstrate its superiority or differential efficiency over other formulations. Accordingly, no comparative advantages are implied, and future multi-source trials are warranted to establish these distinctions. Finally, regarding microbial ecology methodology, we acknowledge that amplicon sequence variant (ASV) approaches (such as DADA2 or Deblur) have become the contemporary standard due to their superior taxonomic resolution and reproducibility compared to traditional operational taxonomic unit (OTU) clustering. In this study, sequence processing was performed using the UPARSE pipeline at a 97% similarity threshold to maintain consistency with historical benchmarks and legacy datasets in this research field [57]. Nevertheless, we recognize that this 97% OTU approach may limit fine-scale species-level resolution and potentially mask micro-diversity within specific taxa, a technical constraint that should be factored into the final interpretation of the microbial data.

## 5. Conclusions

In conclusion, dietary HMSeBA supplementation enhanced selenium bioavailability in blood and milk, strengthened systemic antioxidant defenses, and reduced milk somatic cell count, without compromising lactation performance in cows fed a selenium-adequate basal diet. At the multi-omics level, this supplementation was linked to coordinated shifts in the rumen microbiota and metabolome, characterized by the enrichment of fiber-degrading bacterial genera and concomitant alterations in feed-derived flavonoids, peptide dynamics, and microbial metabolic intermediates. Correlation analysis highlighted the ruminal quorum-sensing signal 3-OH-C6-HSL as a key node positively associated with both fiber-degrading taxa and host blood selenium concentrations, suggesting a potential crosstalk between the ruminal microenvironment and systemic selenium status. Nevertheless, given the limited sample size and exploratory nature of these multi-omics associations, the underlying causal mechanisms require targeted experimental validation in future studies. Overall, HMSeBA represents a promising nutritional intervention to support host antioxidant-immune status and safeguard udder health in dairy cows, warranting further mechanistic investigation.

## Figures and Tables

**Table 1:** Composition and nutrient content in the basal diet (%, DM basis).

Item	Amount
Ingredients	
Alfalfa hay	7.53
Concentrated feed	5.05
Yeast culture	38.95
Premixed ^1^	2.24
Flaked corn	8.02
Cottonseed	4.96
Beet pulp	4.85
Brewers grains	2.43
Silage corn	25.97
Nutrient level	
DM	54.32
Crude protein	12.88
Ether extract	5.71
NDF ^2^	35.86
ADF ^3^	19.18
Ash	7.60
Calcium	0.52
Phosphorus	0.23

^1^ provided as per kilogram of premix: VA 70 000 IU, VD 23 000 IU, VE 380 IU, Fe 109 mg, Cu 115 mg, Zn 628 mg, Co 9 mg, Mn 637 mg, Mg 656 mg. ^2^ NDF, neutral detergent fiber. ^3^ ADF, acid detergent fiber.

**Table 2:** Effects of HMSeBA supplementation on milk yield and dry matter intake (DMI) in dairy cows.

Item	Groups		SEM	*p*-Value
	CG	EG		
Milk yield, kg	30.00	31.20	3.165	0.421
DMI, kg	24.14	22.46	0.100	0.075
MPE	1.26	1.43	0.080	0.134

**Table 3:** Effects of HMSeBA supplementation on blood selenium concentration in dairy cows.

Item	Time/d	Groups		SEM	*p*-Value
		CG	EG		
Blood Se, μg/L	0	57.74	55.22	1.864	0.754
	105	68.95 ^b^	90.29 ^a^	1.385	0.020

^a,b^ values in the same row with different superscripts differ significantly. Statistical significance was set at *p* < 0.05.

**Table 4:** Effects of HMSeBA supplementation on rumen fermentation parameters in dairy cows.

Item	Groups		SEM	*p*-Value
	CG	EG		
pH	6.82	6.64	0.04	0.205
MCP, mg/mL	0.58	0.82	0.03	0.058
NH3-N, mg/dL	13.89	12.5	1.04	0.685
Acetate, mmol/L	47.09	59.12	1.66	0.052
Propionate, mmol/L	21.3	25.73	0.99	0.200
Butyrate, mmol/L	11.00 ^b^	14.51 ^a^	0.43	0.035
Isovaleric acid, mmol/L	0.99	1.42	0.07	0.077
Valeric acid, mmol/L	1.86	1.56	0.31	0.673
Volatile fatty acid, mmol/L	82.27	102.33	2.99	0.067

^a,b^ values in the same row with different superscripts differ significantly. Statistical significance was set at *p* < 0.05.

## Data Availability

The raw sequencing data presented in this study have been deposited in the Genome Sequence Archive (GSA) at the China National Center for Bioinformation (CNCB-NGDC) under BioProject accession number PRJCA065471 (submission ID: subPRO095541). Other original contributions presented in this study are included in the article; further inquiries can be directed to the corresponding author.

## Supplementary Materials

The following supporting information can be downloaded at https://www.mdpi.com/article/10.3390/antiox15070813/s1, Table S1: Representativeness validation of the selected rumen-sampling subset; Table S2: Summary of metabolite selection criteria and filtering steps; Table S3: Differential metabolites identified in ruminal fluid between the CG and EG (VIP > 1, *p* < 0.05, and FDR < 0.1).

## Abbreviations

The following abbreviations are used in this manuscript:

ALB	Albumin
CG	Control group
DM	Dry matter
DMI	Dry matter intake
EG	Experimental group
GLB	Globulin
GSH-Px	Glutathione peroxidase
HMSeBA	Hydroxy-selenomethionine
IgA	Immunoglobulin A
IgG	Immunoglobulin G
IgM	Immunoglobulin M
MCP	Microbial crude protein
MDA	Malondialdehyde
NASEM	The National Academies of Sciences, Engineering and Medicine
OTU	Operational taxonomic unit
PCoA	Principal coordinate analysis
PLS-DA	Partial least squares discriminant analysis
SCC	Somatic cell count
SOD	Superoxide dismutase
TMR	Total mixed ration
TP	Total protein
VFA	Volatile fatty acid
VIP	Variable importance in projection
