# Supplementary material for: Dietary Hydroxy-Selenomethionine Improves Antioxidant Status and Reduces Somatic Cell Count in Dairy Cows: Multi-Omics Insights into Rumen Microbiota and Metabolic Profiles
Source: Antioxidants (Basel). 2026 Jun 28;15(7):813. doi: 10.3390/antiox15070813 (PMC13405601; doi:10.3390/antiox15070813)
Supplement: Supplementary file 1 [file antioxidants-15-00813-s001.zip › antioxidants-4372055-supplementary.pdf]

Supplementary Materials

Table S1. Representativeness validation of the selected rumen-sampling subset.

Item	Control Group			Experimental Group		
	Full (N=20)	Subset (N=5)	*p*-value	Full (N=20)	Subset (N=5)	*p*-value
Parity	1.25 ± 0.14	1.40 ± 0.40	0.668	1.25 ± 0.14	1.20 ± 0.20	0.871
Days in Milk, d	254.75 ± 9.27	231.40 ± 9.48	0.240	251.65 ± 9.70	257.60 ± 19.47	0.786
SCC, $\times 10^4 \cdot mL^{-1}$	14.42 ± 4.74	12.25 ± 6.60	0.816	18.25 ± 5.86	14.40 ± 6.36	0.734
Blood Se, μg/L	57.74 ± 4.24	52.17 ± 4.49	0.528	55.22 ± 7.19	52.42 ± 8.49	0.838
Milk Se, μg/L	44.05 ± 1.72	44.94 ± 3.24	0.811	48.89 ± 2.25	48.56 ± 1.26	0.942

Table S2. Summary of metabolite selection criteria and filtering steps.

Item	Number
Identified metabolites	2618
After confidence filtering[1]	1411
VIP > 1， $p < 0.05$[2]	574
FDR < 0.1[3]	44
FDR < 0.05	17

[1]Confidence filtering retained metabolites with annotations at identification level B(i) or B(ii) (MSI Level 2), corresponding to MS/MS spectral matching against experimental or theoretical reference libraries; metabolites lacking MS/MS support or carrying biologically implausible annotations were excluded.

[2]VIP from OPLS-DA model; p-value from two-tailed Student's t-test.

[3]FDR correction was performed using the Benjamini-Hochberg procedure applied to *p*-values across all 1411 confidence-filtered metabolites. VIP > 1, $p < 0.05$, and FDR < 0.1 were applied as joint criteria. FDR < 0.1, rather than the more conventional < 0.05, was adopted given the exploratory nature of this study and the small sample size ($n = 5$ per group); the more stringent FDR < 0.05 threshold is provided for reference.

Table S3. Differential metabolites identified in ruminal fluid between the CG and EG (VIP > 1, $p < 0.05$, and FDR < 0.1).

Metabolite	VIP	*p*-value	FDR	FC	Regulate	level
Gibberellin A36	5.92	0.000	0.041	3.21	down	B(ii)
Villol	5.08	0.001	0.089	3.49	down	B(i)
2-Hydroxy-6-[(8Z,11Z)-Pentadeca-8,11-Dienyl]Benzoic Acid	3.93	< 0.001	0.006	1.48	down	B(i)
Pc(P-16:0/2:0)	3.50	< 0.001	0.009	0.81	up	B(i)
O6-Carboxymethyl-2'-Deoxy-Guanosine	3.46	< 0.001	0.002	1.20	down	B(ii)
1-[(2R,4R,5R)-5-(Aminomethyl)-4-Hydroxyoxolan-2-Yl]-5-Methylpyrimidine-2,4-Dione	3.37	< 0.001	0.003	1.31	down	B(ii)
6-Methylsulfinylhexanenitrile	3.31	0.000	0.067	1.28	down	B(i)
1,4-Anhydro-5-(Benzoylamino)-2,5-Dideoxy-2-(4-Piperidinylamino)-D-Arabinitol	3.23	0.000	0.071	1.24	down	B(i)
5-Methyldeoxycytidine	3.21	< 0.001	0.011	1.31	down	B(ii)
Luteolin 7-O-(6''-O-Malonyl)-Beta-D-	3.10	< 0.001	0.004	1.26	down	B(ii)

Diglucoside						
Tyr-Val-Ile	3.04	0.000	0.037	1.27	down	B(i)
His-His	2.99	0.001	0.082	1.19	down	B(i)
Zeanic Acid	2.98	< 0.001	0.007	1.21	down	B(ii)
Xanthotoxol	2.78	0.000	0.055	1.18	down	B(i)
Furo[3,2-C]Pyran-4-One	2.61	< 0.001	0.014	0.84	up	B(ii)
Phe-Ser-Trp	2.61	0.000	0.051	1.11	down	B(i)
8-Nitroguanine	2.60	0.001	0.089	1.21	down	B(ii)
3-Hydroxy-Hexanoyl-Dl-Homoserine Lactone	2.57	0.000	0.055	0.86	up	B(i)
Cabbage Identification Factor 2	2.44	< 0.001	0.022	1.11	down	B(ii)
3-Methyl-N-(1-Methyl-2-Oxo-3,4-Dihydroquinolin-6-Yl)Thiophene-2-Carboxamide	2.35	0.000	0.060	1.09	down	B(i)
N-Methylcaprolactam	2.34	0.001	0.084	1.12	down	B(i)
Linalool (8-Hydroxydihydro-)	2.33	0.001	0.089	0.88	up	B(ii)
2-Piperidone	2.33	< 0.001	0.037	1.08	down	B(i)
Glycylprolylhydroxyproline	2.32	< 0.001	0.003	0.88	up	B(ii)
Flindersine	2.28	0.001	0.084	1.08	down	B(i)
3-(2,6-Dihydroxyphenyl)-4-Hydroxy-6-Methyl-3H-2-Benzofuran-1-One	2.25	0.001	0.089	0.90	up	B(i)
3-Phenyl-3-(Pyridin-2-Yl)Propanoic Acid	2.25	0.000	0.064	1.09	down	B(i)
Ala Met Arg	2.23	0.000	0.055	0.90	up	B(i)
Catechin 7-Glucoside	2.22	< 0.001	0.004	0.90	up	B(ii)
1-Deoxy-D-Xylulose-5-Phosphate	2.21	0.001	0.088	0.87	up	B(i)
3-Methylidene-4-[3-Methyl-4-(4-Methyl-5-Oxooxolan-2-Yl)But-2-Enyl]Oxolan-2-One	2.17	0.000	0.055	1.09	down	B(i)
1-Hexanol Arabinosylglucoside	2.16	< 0.001	0.013	0.90	up	B(ii)
Salicyluric Acid	2.15	0.000	0.041	1.10	down	B(ii)
N-Palmitoyl Gaba	2.08	0.000	0.061	1.08	down	B(ii)
2-Amino-7-Methoxy-3H-Phenoxazin-3-One	2.08	0.000	0.032	1.11	down	B(ii)
4-Acetoxy Met	2.04	0.001	0.080	0.93	up	B(i)
5-Hydroxyindol-2-Carboxylic Acid	2.01	0.000	0.008	1.08	down	B(i)
2-Methyl-6-[(2,2,2-Trifluoroacetyl)Amino]Benzoic Acid	1.97	0.000	0.027	1.10	down	B(i)
Gln-Val-His	1.88	0.000	0.019	0.93	up	B(i)
Gartanin	1.82	0.001	0.095	0.93	up	B(ii)
Epinepetalactone	1.80	0.001	0.084	1.07	down	B(i)
7-Methylguanosine	1.78	0.001	0.076	1.09	down	B(ii)
Thymyl Acetate	1.29	0.000	0.055	1.03	down	B(i)
Scopolin	1.05	0.001	0.072	0.98	up	B(ii)
